# Perioperative antithrombotic management of patients who receive direct oral anticoagulants during gastroenterological surgery

**DOI:** 10.1002/ags3.12328

**Published:** 2020-04-01

**Authors:** Takahisa Fujikawa, Ryo Takahashi, Shigetoshi Naito

**Affiliations:** ^1^ Department of Surgery Kokura Memorial Hospital Kitakyushu Japan

**Keywords:** anticoagulation therapy, bleeding complication, direct oral anticoagulant, gastroenterological surgery, thromboembolic complication

## Abstract

**Aim:**

We investigated the effect of perioperative management of direct oral anticoagulants (DOACs) on bleeding and thromboembolic complications during gastroenterological (GE) surgery.

**Methods:**

A total of 334 patients receiving anticoagulants and undergoing elective GE surgery between 2012 and 2018 were enrolled. The patients were divided into three groups: patients receiving warfarin (WF, n = 231), patients receiving DOACs with heparin bridging (DOAC‐HB, n = 34), and patients receiving DOAC without heparin bridging (DOAC‐NHB, n = 69). Outcome variables were compared between the groups and the risk factors of postoperative bleeding were assessed using logistic multivariate analysis.

**Results:**

No significant differences were observed in background characteristics between the groups. There were similarities between the groups in surgical blood loss (*P* = .772) and rate of intraoperative transfusion (*P* = .952). Thromboembolic complications only occurred in two patients in the WF group (0.9%), and no thromboembolism occurred in the DOAC groups. The incidence of major postoperative bleeding was significantly higher in DOAC‐HB group than in the other groups (14.7% vs 4.8% vs 1.4%, *P* = .011). Multivariate analysis showed DOAC with heparin bridging to be the most significant risk factor of major postoperative bleeding (odds ratio = 11.60, *P* = .028).

**Conclusions:**

Elective GE surgery can be safely performed in patients receiving DOACs without heparin bridging. Perioperative heparin bridging during DOAC interruption is not recommended even for patients undergoing major GE surgery due to increased postoperative bleeding.

## Graphical abstract

In a multivariate analysis of 334 anticoagulant‐received patients undergoing elective gastroenterological surgery, it is demonstrated that direct oral anticoagulant (DOAC) combined with heparin bridging was the most significant risk factor for postoperative bleeding complication. The importance of this finding is that perioperative bridging with heparin is not recommended during DOAC therapy interruption even when major gastroenterological surgery is performed.

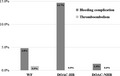

## 1 INTRODUCTION

The number of patients who receive anticoagulation therapy (ACT) for the prevention of ischemic stroke or venous thromboembolism is increasing with the aging of the population. Since clinical trials showed non‐inferiority or superiority of direct oral anticoagulant (DOAC) therapy over vitamin K antagonist (warfarin) therapy in terms of safety and efficacy for patients with atrial fibrillation,^1^, ^2^, ^3^, ^4^, ^5^ the number of patients who receive DOAC therapy has been increasing. Recent reports also indicate that approximately 10%–15% of patients treated with DOACs have to interrupt their anticoagulant treatment before an invasive procedure every year.^6^, ^7^

Direct oral anticoagulants, also known as non‐vitamin K antagonist oral anticoagulants (NOACs), include direct thrombin inhibitors, such as dabigatran, and factor Xa inhibitors, such as rivaroxaban, apixaban, and edoxaban. The potential advantages of DOACs over warfarin include rapid onset and offset of action, reduced effect of dietary vitamin K intake or drug interaction on their activity, and predictable anticoagulant effects with no need for routine monitoring.^5^, ^8^ Although updated guidelines on digestive endoscopic procedures indicate the optimal perioperative management for patients who receive DOAC treatment,^9^, ^10^ perioperative management during gastroenterological (GE) surgery, which is considered to carry a high risk of bleeding, is yet to be established as it remains a challenge. In this study, we reviewed 334 consecutive patients who received ACT and underwent elective GE surgery, and we investigated the effect of perioperative management with DOACs on bleeding and thromboembolic complications.

## 2 METHODS

### 2.1 Patients

**Figure 1:**
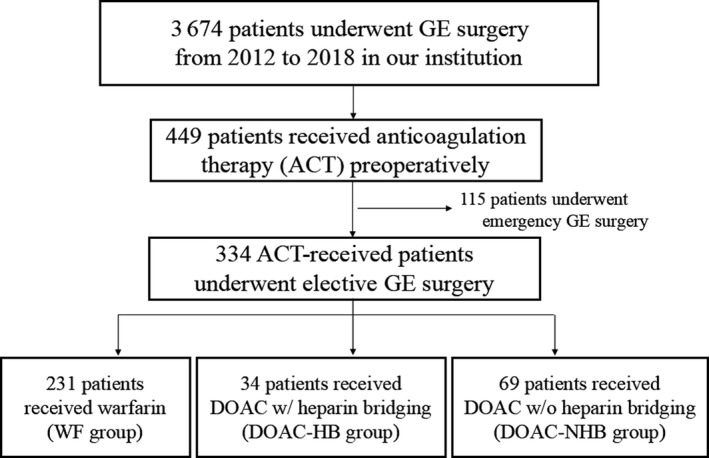
Consort diagram of the study. Abbreviations: ACT, anticoagulation therapy; DOAC, direct oral anticoagulant; GE, gastroenterological; HB, heparin bridging; NHB, non‐heparin bridging; WF, warfarin

Our institutional review board approved this study (#19061903). We searched the prospectively collected surgery database of a single institution for relevant cases, and we included 334 consecutive patients who underwent GE surgery in this study and excluded patients who underwent emergency surgery (Figure 1). The cohort included 240 patients with malignant disease and 94 patients with benign disease, and, in terms of the mode of surgery, the cohort included 200 patients who underwent laparoscopic surgery and 134 patients who underwent open surgery. Based on the type of perioperative ACT, the patients were divided into three groups: patients who received warfarin therapy (WF group, n = 231), patients who received DOAC therapy with heparin bridging (DOAC‐HB group, n = 34), and patients who received DOAC therapy without heparin bridging (DOAC‐NHB group, n = 69). All procedures were performed by or under the guidance of one of the board‐certified attending surgeons in our institution. The choice of perioperative ACT was based on patient condition and risk of thromboembolism. The management of patients with high thromboembolic risk included interruption of oral ACT, bridging anticoagulation with unfractionated heparin, and early postoperative reinstitution of oral ACT. In case of warfarin‐received patients, warfarin was interrupted 5 days before the operation, heparin was started and continued until the day of surgery, warfarin and postoperative bridging with heparin were resumed on postoperative day (POD) 1–2 when hemostasis was secured, and discontinued bridging when the PT‐INR was within therapeutic range. In case of DOAC‐received patients with bridging heparin, DOAC was interrupted 1–3 days before the operation, heparin was started and continued until the day of surgery, DOACs and heparin bridging were resumed on POD 1–2, and heparin was discontinued on POD 4–5. If DOAC therapy was managed without bridging heparin, DOAC was stopped 0–1 days before the surgery and resumed 1–2 days after the surgery when hemostasis was secured.

If a patient received antiplatelet therapy such as aspirin or clopidogrel, the perioperative management was performed according to the previously described perioperative management protocol (the Kokura Protocol).^11^, ^12^ Generally, antiplatelet agents were discontinued one week before surgery, but in patients with high thromboembolic risks, preoperative aspirin monotherapy was continued until the day before surgery. Postoperatively, early reinstitution of antiplatelets was performed unless there were signs of bleeding. For prevention of venous thromboembolism, mechanical prophylaxis (intermittent pneumatic compression and/or graduated compression stockings) and enforcement of early postoperative walking were generally performed, although routine use of medical prophylaxis with heparin was not adopted, except in the case of high venous thromboembolic risk patients with previous venous thrombosis or immobilization.

To assess the predicted thromboembolic risk of patients in each group, we used the revised CHADS_2_ scoring system,^13^, ^14^, ^15^ which is widely used for the prediction of ischemic stroke or transient ischemic attack (TIA) in patients with atrial fibrillation or atrial flutter. This scoring system is an assessment tool that evaluates congestive heart failure, hypertension, age, diabetes, and ischemic stroke, and it categorizes patients with a score of 2 or higher into the high‐risk group. It has been reported that the revised CHADS_2_ scoring system also predicts ischemic stroke and death in patients without a history of atrial fibrillation or atrial flutter.^16^, ^17^ To assess the predicted bleeding risk of patients in each group, we used the HAS‐BLED score,^18^ which is widely used for the predicting bleeding risk in anticoagulated patients with atrial fibrillation.

The severity of patient symptoms and level of patient functioning in terms of ambulation were reported according to the Eastern Cooperative Oncology Group scale of performance status.^19^ Postoperative complications were assessed and categorized according to the Clavien‐Dindo classification (CDC) and CDC class 2 or higher was considered significant.^20^ Postoperative thromboembolic complication was defined as per earlier reports.^11^, ^12^ In brief, thromboembolism included cerebral infarction, myocardial infarction, mesenteric infarction, pulmonary thromboembolism, and acute arterial embolism. Postoperative bleeding complication was categorized by ISTH (International Society of Thrombosis and Haemostasis) definition,^21^ which included luminal bleeding (for example, gastrointestinal bleeding), abdominal bleeding, intracranial hemorrhage, and abdominal wall hematoma. Operative mortality was defined as death within 30 days after surgery.

The primary outcome included intraoperative blood loss and postoperative bleeding complications. The background characteristics, perioperative factors, and surgical outcomes of the included patients were compared between the groups, and the risk factors of postoperative bleeding complication were assessed using logistic multivariate analysis.

### 2.2 Statistical analysis

Continuous values were expressed as mean (standard deviation) or median (interquartile range), while categorical variables were presented as absolute numbers and percentages. For univariate comparisons, Fisher's exact probability test was used to evaluate categorical variables, and continuous variables were analyzed using one‐way analysis of variance and Kruskal‐Wallis test for normally distributed data and non‐normally distributed data, respectively. Multivariate logistic regression analysis was performed to determine the risk factors of thromboembolic complications. All *P*‐values were two‐sided, and *P*‐values less than .05 were considered statistically significant. All statistical analyses were performed using EZR (Saitama Medical Centre, Jichi Medical University), which is a graphical user interface for R (The R Foundation for Statistical Computing, Vienna, Austria, version 2.13.0).^22^

## 3 RESULTS

### 3.1 Patient and operative characteristics

**Figure 2:**
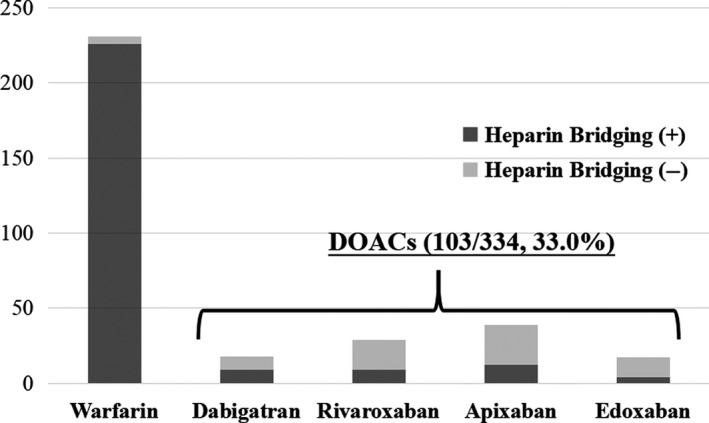
Types of anticoagulation agents administered to the cohort. Warfarin was the most commonly used agent and was administered to 67% of patients, whereas DOACs were administered to 33% of patients. A total of 226 of the 231 patients (97.8%) who were administered warfarin also received perioperative management with heparin bridging, but only 34 of the 103 patients (33.0%) who were administered DOACs also received perioperative management with heparin bridging. Abbreviation: DOAC, direct oral anticoagulant

A total of 334 patients were enrolled in this study. Figure 2 shows the types of anticoagulation agents administered to this cohort. Warfarin was the most commonly used agent and was administered to 231 of the 334 patients (69.2%), whereas DOAC was administered to 103 of the 334 patients (30.8%). Most patients in the WF group, a total of 226 out of 231 patients (97.8%), were perioperatively managed with heparin bridging. Of the 103 patients who received DOACs, 18, 29, 39, and 17 of them were administered dabigatran, rivaroxaban, apixaban, and edoxaban, respectively, and only 34 of the 103 patients (33.0%) received perioperative heparin bridging.

Table 1 shows the background characteristics of each group. The median ages in the WF, DOAC‐HB, and DOAC‐NHB groups were 75 years, 75 years, and 74 years, respectively (*P* = .927). There were also similarities between the groups in poor performance status of patients (grades 2–4) (*P* = .318), hypertension (*P* = .265), diabetes mellitus (*P* = .167), vascular diseases (*P* = .492), history of congestive heart failure (*P* = .116), abnormal liver function (*P* = .931), and history of cerebral infarction or TIA (*P* = .435) except for abnormal liver function (10.0% vs 0.0% vs 1.4%, *P* < .001). Concerning indication for ACT, non‐valvular atrial fibrillation or flutter is the most prevalent in the whole cohort, and there was a difference in the rates of non‐valvular (61.9% vs 79.4% vs 84.1%, *P* = .002) and valvular atrial fibrillation/flutter (10.8% vs 5.9% vs 0.0%, *P* = .030) between the groups. The rates of patients receiving antiplatelet therapy and those treated by preoperative continuation of aspirin were identical between the groups. There was also no difference between the groups in the occurrence of patients with high bleeding risks categorized by HAS‐BLED score (44.6% vs 38.2% vs 37.7%, *P* = .634). The percentages of patients in the high‐risk category according to the revised CHADS_2_ scoring system in the WF, DOAC‐HB, and DOAC‐NHB groups were 57.6%, 61.8%, and 59.4%, respectively (*P* = .606).

**Table 1:** Background characteristics of patients in the current cohort

Variables	WF (n = 231)	DOAC‐HB (n = 34)	DOAC‐NHB (n = 69)	*P* value
Age, y, median (range)	75 [44‐94]	75 [51‐87]	74 [44‐88]	.927
Gender, n (%)				
Female	71 (30.7)	9 (26.5)	23 (33.3)	.777
Male	160 (69.3)	25 (73.5)	46 (66.7)	
BMI, kg/m^2^, median (range)	22.90 [14.2‐32.4]	23.6 [18.3‐33.3]	23.8 [14.2‐36.7]	.087
Performance status, n (%)				
0, 1	194 (84.0)	32 (94.1)	59 (85.5)	.318
2‐4	37 (16.0)	2 (5.9)	10 (14.5)	
Concurrent diseases, n (%)				
Hypertension	91 (39.6)	12 (35.3)	34 (49.3)	.272
Diabetes mellitus	51 (22.2)	6 (17.6)	22 (31.9)	.170
Hx of congestive heart failure	85 (36.8)	7 (20.6)	20 (29.0)	.127
Vascular diseases	62 (27.0)	6 (17.6)	19 (27.5)	.492
Hx of cerebral infarction/TIA	48 (20.8)	8 (23.5)	10 (14.5)	.454
Abnormal renal function	**23 (10.0)**	**0 (0.0)**	**1 (1.4)**	**<.001**
Abnormal liver function	19 (8.2)	2 (5.9)	7 (10.1)	.931
Hx of bleeding	67 (29.0)	13 (38.2)	21 (30.4)	.670
Indication for ACT, n (%)				
AF (non‐valvular)	**143 (61.9)**	**27 (79.4)**	**57 (82.6)**	**.002**
AF (valvular)	**25 (10.8)**	**2 (5.9)**	**0 (0.0)**	**.030**
s/p cardiac valve replacement	34 (14.7)	1 (2.9)	0 (0.0)	.105
Hx of thrombosis	23 (10.0)	3 (8.8)	5 (7.2)	.906
Antiplatelet therapy, n (%)	107 (46.5)	11 (32.4)	23 (33.3)	.070
Continued antiplatelet thearpy, n (%)	53 (22.9)	8 (23.5)	19 (27.5)	.734
HAS‐BLED score, n (%)				
Score 0‐2	128 (55.4)	21 (61.8)	43 (62.3)	.634
Score 3 or higher	103 (44.6)	13 (38.2)	26 (37.7)	
CHADS_2_ score, n (%)				
Score 0	21 (9.1)	5 (14.7)	7 (10.1)	.606
Score 1	77 (33.3)	8 (23.5)	21 (30.4)	
Score 2 or higher	133 (57.6)	21 (61.8)	41 (59.4)	

Note

Bold value indicates statistically significant.

Abbreviations: ACT, anticoagulation therapy; AF, atrial fibrillation or flutter; BMI, body mass index; DOAC, direct oral anticoagulant; Hx, history; TIA, transient ischemic attack; WF, warfarin.

Table 2 shows the factors associated with the operative procedures in the whole cohort. The cohort consisted of 94 patients with benign disease and 240 patients with malignant disease, and 191 esophagogastrointestinal resections and 113 hepatobiliary and pancreatic resections were performed. In terms of the mode of surgery, 200 patients underwent laparoscopic surgery, and 134 patients underwent open surgery. There were no statistically significant differences between the groups in the type of disease (*P* = .373) and type of surgery (*P* = .952), but laparoscopic surgery was less frequently performed in the WF group than in the DOAC‐HB group and the DOAC‐NHB group (54.5% vs 67.6% vs 73.9%, *P* = .009). Although there were similarities in surgical blood loss (*P* = .772) and rate of intraoperative red blood cell (RBC) transfusion (*P* = .919) between the groups, the duration of operation was shorter in the WF group than in the DOAC‐HB group and the DOAC‐NHB group (177 minutes vs 195 minutes vs 222 minutes, *P* = .035).

**Table 2:** Factors regarding operative procedures in the cohort

Variables	WF (n = 231)	DOAC‐HB (n = 34)	DOAC‐NHB (n = 69)	*P* value
Type of diseases, n (%)				
Benign diseases	70 (30.3)	7 (20.6)	17 (24.6)	.373
Malignant diseases	161 (69.7)	27 (79.4)	52 (75.4)	
Type of surgery, n (%)				
GI surgery				
Esophagogastric resection	42 (18.2)	6 (17.6)	16 (23.1)	.952
Colorectal resection	87 (37.7)	15 (44.1)	25 (36.2)	
HBP surgery				
Cholecystectomy	45 (19.5)	6 (17.6)	13 (18.8)	
Liver resection	24 (10.4)	3 (8.8)	7 (10.1)	
Panreatic resection	9 (3.9)	2 (5.9)	4 (5.8)	
Others	24 (10.4)	2 (5.9)	4 (5.8)	
Mode of surgery, n (%)				
Open surgery	**105 (45.5)**	**11 (32.4)**	**18 (26.1)**	**.009**
Laparoscopic surgery	**126 (54.5)**	**23 (67.6)**	**51 (73.9)**	
Duration of operation, min, median (range)	**177 [48‐657]**	**194.5 [68**‐**659]**	**222 [33**‐**645]**	**.035**
Surgical blood loss, mL, median (range)	35 [0‐3400]	35 [0‐2100]	35 [0‐1040]	.772
Surgical blood loss ≥ 500 mL, n (%)	19 (8.2)	3 (8.8)	5 (7.2)	.952
Intraoperative RBC transfusion, n (%)	18 (7.8)	2 (5.9)	5 (7.2)	.919

Note

Bold value indicates statistically significant.

Abbreviations: DOAC, direct oral anticoagulant; RBC, red blood cell; WF, warfarin.

### 3.2 Postoperative bleeding and thromboembolic complications

Table 3 shows the factors associated with postoperative morbidity and mortality in the cohort. Postoperative complications occurred in 95 of the 334 patients (28.4%). The incidences of severe postoperative complications (CDC class 3 or higher) in the WF, DOAC‐HB, and DOAC‐NHB groups were 8.7%, 20.6%, and 5.8%, respectively (*P* = .071). Thromboembolic complications were observed in only two of the 231 patients (0.9%) in the WF group (cerebral infarction in one patient and pulmonary embolism in the other), but thromboembolism did not occur in any patient treated with a DOAC.

**Table 3:** Factors concerning postoperative morbidity and mortality in the cohort

Variables	WF (n = 231)	DOAC‐HB (n = 34)	DOAC‐NHB (n = 69)	*P* value
Postoperative complication, n (%)				
C‐D class 0	166 (71.9)	18 (52.9)	55 (79.7)	.071
C‐D class 1	13 (5.6)	3 (8.8)	6 (8.7)	
C‐D class 2	32 (13.9)	6 (17.6)	4 (5.8)	
C‐D class 3 or higher	20 (8.7)	7 (20.6)	4 (5.8)	
Postop. bleeding complication, n (%)	**11 (4.8)**	**5 (14.7)**	**1 (1.4)**	**.011**
Luminal bleeding, n (%)	9 (3.9)	3 (8.8)	0 (0.0)	
Abdominal bleeding, n (%)	1 (0.4)	2 (5.9)	0 (0.0)	
Abdominal wall hematoma, n (%)	0 (0.0)	0 (0.0)	1 (1.4)	
Intracranial hemorrhage, n (%)	1 (0.4)	0 (0.0)	0 (0.0)	
Postop. thrombotic complication, n (%)	2 (0.9)	0 (0.0)	0 (0.0)	.637
Cerebral infarction, n (%)	1 (0.4)	0 (0.0)	0 (0.0)	
Pulmonary embolism, n (%)	1 (0.4)	0 (0.0)	0 (0.0)	
Operative mortality, n (%)	3 (1.3)	0 (0.0)	1 (1.4)	.791
Length of postop. stay, d, median (range)	**14 [3‐121]**	**12.5 [3‐64]**	**11 [3‐147]**	**.009**

Note

Bold value indicates statistically significant.

Abbreviations: C‐D, Clavien‐Dindo; DOAC, direct oral anticoagulant; NA, not available; Postop., postoperative; WF, warfarin.

**Figure 3:**
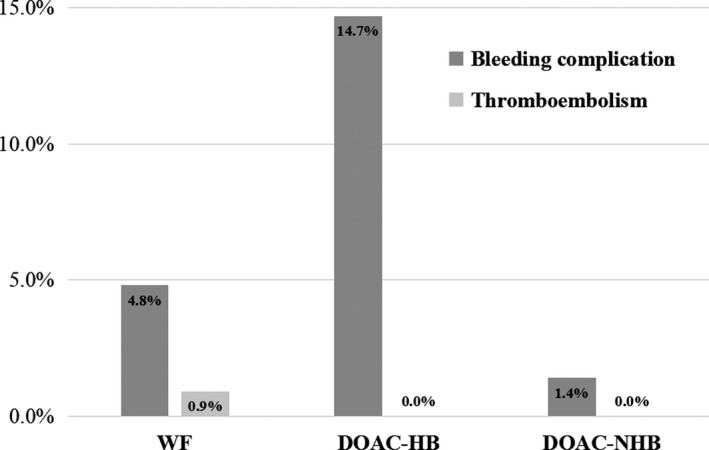
Incidence of postoperative bleeding and thromboembolic complications in each group. Thromboembolic complications only occurred in 2 of the 231 patients (0.9%) in the WF group, but thromboembolism did not occur in the patients treated with DOACs. The incidence of postoperative bleeding complication was significantly higher in the DOAC‐HB group than in the WF group and the DOAC‐NHB group (14.7% vs 4.8% vs 1.4%, *P* = .011). In the DOAC‐NHB group, only I case of postoperative bleeding (abdominal wall hematoma) was observed. Abbreviations: DOAC, direct oral anticoagulant; HB, heparin bridging; NHB, non‐heparin bridging; WF, warfarin

The incidence of major postoperative bleeding complication was significantly higher in the DOAC‐HB group than in the WF group and the DOAC‐NHB group (14.7% vs 4.8% vs 1.4%, *P* = .011, Figure 3). There was only a single case of postoperative bleeding (abdominal wall hematoma) in the DOAC‐NHB group, which occurred on POD 49 after pancreaticoduodenectomy and persistent postoperative pancreatic fistula, in a patient receiving both apixaban and aspirin, and required repeated hemostasis. Postoperative bleeding occurred in five patients in the DOAC‐HB group, which included three with gastrointestinal bleeding requiring endoscopic hemostasis (anastomotic bleeding in two and bleeding from gastric ulcer in one) and two with intra‐abdominal bleeding requiring relaparotomy. All five bleeding complications occurred after gastrointestinal cancer surgery with anastomosis, and four out of five cases occurred during heparin bridging period (POD 3‐5), except for the case of bleeding from gastric Dieulafoy ulcer, which occurred on POD 23 after persistent postoperative ileus. The mortality rates in the WF, DOAC‐HB, and DOAC‐NHB groups were 1.3%, 0.0%, and 1.4%, respectively (*P* = .791).

### 3.3 Risk factors affecting thromboembolic and bleeding complications

Univariate and multivariate analyses of postoperative bleeding complications in the cohort were conducted and are shown in Table 4. Univariate analysis revealed that only DOAC treatment with heparin bridging is associated with postoperative bleeding. Multivariate analysis also showed that DOAC treatment with heparin bridging is independently and significantly associated with postoperative bleeding (odds ratio = 11.60, *P* = .028).

**Table 4:** Univariate and multivariate analyses of postoperative bleeding complication in the whole cohort (n = 334)

Variables	Univariate	Multivariate analyses		
	*P* value	Odds ratio	95% CI	*P* value
Age ≥ 75 y	.451	—	—	—
Male gender	.784	—	—	—
BMI ≥ 30 kg/m^2^	1.000	—	—	—
Performance status 2‐4	.712	—	—	—
Hypertension	.450	—	—	—
Diabetes mellitus	.771	—	—	—
Vascular diseases	.771	—	—	—
History of CHF	.280	—	—	—
History of cerebral infarction/TIA	.747	—	—	—
Preoperative aspirin continuation	.549	1.460	0.49‐4.38	.500
Warfarin therapy	.611	3.130	0.38‐25.0	.290
DOAC with heparin bridging	**.011**	**11.60**	**1.29**‐**104.0**	**.028**
HBP surgery	.480	—	—	—
Laparoscopic surgery	.199	0.560	0.20‐1.54	.260

Note

Bold value indicates statistically significant.

Abbreviations: APT, antiplatelet therapy; BMI, body mass index; CHF, congestive heart failure; CI, confidence interval; DOAC, direct oral anticoagulant; HBP, hepatobiliary and pancreas; TIA, transient ischemic attack.

## 4 DISCUSSION

In this study, which reviewed 334 patients who received ACT and underwent elective GE surgery, it was found that the incidences of overall postoperative complication, major bleeding complication, and thromboembolism were 28.4%, 5.1%, and 0.6%, respectively. Thirty‐four patients (33.0%) treated with DOACs received perioperative heparin bridging. Surgical blood loss and rate of RBC transfusion were identical between the groups, and thromboembolism was observed only in the WF group (0.9% of patients in the WF group). The incidence of major postoperative bleeding was significantly higher in the DOAC‐HB group than in the other groups, and multivariate analysis showed that DOAC therapy with heparin bridging is the most significant risk factor of major postoperative bleeding (odds ratio = 11.60, *P* = .028). Therefore, perioperative bridging with heparin is not recommended during DOAC therapy interruption even for patients who undergo major GE surgery.

Currently, DOACs are increasingly prescribed for the prevention of arterial or venous thromboembolism. In 2011, dabigatran was approved and is indicated for the prevention of ischemic stroke and systemic embolism in patients with non‐valvular atrial fibrillation. Factor Xa inhibitors were then launched with the same indications. They are “easy‐to‐use” drugs with a wide range of safety. Compared to warfarin, DOACs have superior pharmacological properties such as better dose‐response, less difference in anticoagulant activity between individuals, no effect of vitamin K intake on anticoagulant activity, and few drug interactions.^5^, ^23^ Clinically, DOAC therapy also has many advantages including a 19% reduction in mortality and a 52% reduction in the incidence of intracranial hemorrhage compared to warfarin therapy.^5^, ^23^ There is strong evidence from large‐scale randomized controlled trials supporting the use of all four DOAC agents, and the results of a meta‐analysis on these four agents have been published in The Lancet.^5^ The efficacy of DOACs (in preventing thromboembolism) is significantly higher than that of warfarin, and the safety of DOACs (in preventing bleeding events) is similar to that of warfarin.

DOACs are fast‐acting agents that reach peak blood concentration 0.5–5 hours after administration. The half‐lives of DOACs are also short (approximately 12 hours), and their anticoagulant effect fades within 48 hours after their withdrawal.^9^ It has been reported that the anti‐factor Xa activity of factor Xa inhibitors fades within 48 hours of last intake in patients who receive them once a day (for example, rivaroxaban and edoxaban), and it fades within 36 hours in patients who receive them twice a day (for example, apixaban).^24^, ^25^ Therefore, careful assessment of the risks and benefits to patients is necessary when DOAC therapy is stopped for longer than 36–48 hours.

The recently updated guideline on GE endoscopy and antithrombotic therapy^9^ recommends discontinuation of DOAC therapy on the morning of the procedure and resumption of DOAC therapy on the morning after the procedure in patients at high risk of bleeding. However, there is no evidence or guideline yet on GE surgery for patients who are administered DOACs; therefore, the safety of surgical procedures, including open GE surgery and laparoscopic GE surgery, should be assessed. This study showed that perioperative management using DOACs without heparin bridging is safe and feasible even for patients who undergo GE surgery.

With regard to the perioperative management of patients who received DOAC therapy, heparin replacement after cessation of DOAC therapy was initially recommended in the guidelines for gastrointestinal endoscopy or non‐cardiac surgery.^26^, ^27^ However, the therapeutic effect of DOACs diminishes shortly after onset of action, and the major adverse effects of heparin replacement (including increased postoperative bleeding) were identified in the BRIDGE study or in other studies.^12^, ^28^ The most recently updated guidelines recommend DOAC withdrawal for only a short period (36–48 hours) during the surgery and procedure but did not recommend heparin bridging during DOAC cessation even for procedures associated with a high risk of bleeding.^9^, ^29^, ^30^

Several reviews and large‐scale cohort studies^31^, ^32^ also suggest that perioperative management of patients using DOACs without heparin bridging is safe and feasible during non‐cardiac surgery. A recently published prospective multicenter cohort study (the PAUSE study) examined outcomes in 3007 adult patients with atrial fibrillation who received DOAC therapy and underwent an elective surgery or procedure.^32^ DOAC therapy was interrupted 1–2 days prior and resumed 1–2 days after the surgery or procedure. The rate of major bleeding 30 days after the surgery or procedure was 0.90%–1.85%, and the rate of arterial thromboembolism was 0.16%–0.60%. The study suggested that a standardized perioperative DOAC management strategy without heparin bridging can be safely used for patients with atrial fibrillation who undergo surgery. Although the patients in this study underwent a relatively small number of major digestive surgeries, the optimal DOAC therapy without heparin bridging is recommended even for patients who undergo surgeries with a high risk of bleeding such as GE surgery. Our study also showed that perioperative DOAC management without heparin bridging is safe and feasible even for patients who undergo GE surgery.

In this study, the rate of postoperative bleeding complication was significantly higher in the DOAC‐HB group than in the other groups, and luminal (gastrointestinal) bleeding was the most frequent type of bleeding. Heparin bridging may accelerate the incidence of luminal bleeding especially in patients who undergo GE surgery with gastrointestinal anastomosis. The correlation between DOAC therapy with heparin bridging and postoperative luminal bleeding should be further investigated.

This study has some limitations. First, it is a retrospective review from a single center, and this reduces the strength of the conclusion. This limitation will be mitigated in a later follow‐up study. Second, our institution is a high‐volume tertiary referral hospital for surgical patients who receive antithrombotic therapy; therefore, our findings may not be generalizable to lower‐volume centers. This limitation can be minimized by conducting prospective multi‐institutional studies.

## 5 CONCLUSIONS

This study, in reviewing 334 patients who received ACT and underwent elective GE surgery, showed that DOAC therapy with heparin bridging is the most significant risk factor for postoperative bleeding complication. Perioperative bridging with heparin is not recommended during DOAC therapy interruption even for patients who undergo major GE surgery.

## DISCLOSURES

Conflicts of Interest: All authors declare no conflicts of interest.

Ethical Approval: This work was approved by the ethics committee of Kokura Memorial Hospital (#19061903).

Author Contributions: TF: study conception and design. TF, RT and SN: acquisition of data. TF: analysis and interpretation of data; drafting of manuscript. TF and SN: critical revision.
